# Psychometric Properties of the Chinese Shortened Version of the Zuckerman–Kuhlman Personality Questionnaire in a Sample of Adolescents and Young Adults

**DOI:** 10.3389/fpsyg.2017.00349

**Published:** 2017-03-07

**Authors:** Daoyang Wang, Mingming Hu, Chanjin Zheng, Zhengguang Liu

**Affiliations:** ^1^Collaborative Innovation Center of Assessment toward Basic Education Quality, Beijing Normal UniversityBeijing, China; ^2^Department of Psychology, Anhui Normal UniversityWuhu, China; ^3^State Key Laboratory of Cognitive Neuroscience and Learning, Institute of Brain and Cognitive Sciences, Beijing Normal UniversityBeijing, China; ^4^School of Psychology, Jiangxi Normal UniversityNanchang, China

**Keywords:** ZKPQ-III-R, reliability, validity, confirmatory factor analysis, adolescents

## Abstract

**Introduction:** The original 89-item Zuckerman–Kuhlman Personality Questionnaire (form III Revised, ZKPQ-III-R) is a widely accepted and used self-report measure for personality traits. This study assessed the reliability and construct validity of the Chinese short 46-item version of the ZKPQ-III-R in a sample of adolescents and young adults.

**Methodology:** A total of 1,019 Chinese adolescents and young adults completed the Chinese version of the original 89-item version ZKPQ-III-R and short 46-item version ZKPQ-III-R, self-report measures of depression, life satisfaction, and subjective health complaints (SHC), the Big Five personality traits, and a substance use risk profile. We explored the internal consistency of five dimensions of the short 46-item version ZKPQ-III-R and compared it with observations in previous studies of Chinese and other populations. The structure of the questionnaire was analyzed by confirmatory factor analysis and exploratory structural equation modeling.

**Results:** The short 46-item version ZKPQ-III-R had adequate internal reliability for all five dimensions, with Cronbach’s α coefficients of 0.63 to 0.84. The concurrent validity of the short 46-item version ZKPQ-III-R was supported by significant correlations with depression, life satisfaction, and SHC. The short 46-item version ZKPQ-III-R had better fit, similar reliability coefficients, and slightly better construct and convergent validity than the 89-item version.

**Conclusion:** The Chinese version of the 46-item ZKPQ-III-R presented reliability and validity in measuring personality in Chinese adolescents and young adults.

## Introduction

The Zuckerman–Kuhlman Personality Questionnaire (form III Revised [ZKPQ-III-R]) was developed to assess the five basic dimensions of the Alternative Five-Factor Model (AFFM) that was proposed by Zuckerman et al. (1993). The AFFM is considered a revised model of the “Big Five,” which consists of five domains of personality. The original version ZKPQ-III-R consisted of 89 items measuring five dimensions: Impulsive Sensation Seeking (ImpSS), Neuroticism-Anxiety (N-Anx), Aggression-Hostility (Agg-Host), Activity (Act), and Sociability (Sy) (Rahim et al., 2013). ImpSS items involve a lack of planning and tendency to act impulsively without thinking, experience seeking, or the willingness to take risks for the sake of excitement or novel experiences. N-Anx items refer to emotional upset, worry, fearfulness, tension, obsessive indecision, a lack of self-confidence, and sensitivity to criticism. Agg-Host items describe a readiness to express verbal aggression, rudeness, thoughtless or antisocial behavior, vengefulness, and spitefulness and refer to having a quick temper and impatience with others. Act items describe the need for Act and an inability to relax and do nothing when the opportunity presents itself or a preference for challenging work, an active lifestyle, and a high level of energy. Sy items describe the number of friends one has and outgoingness at parties and a preference for being with others as opposed to being alone.

The ZKPQ-III-R has exhibited equivalent factor structure and reliability in several different language versions, and the psychometric properties generally indicated that these translations are valid and reliable (Ostendorf and Angleitner, 1994; Wu et al., 2000; Herrero, 2001; De Pascalis and Russo, 2003; Rossier et al., 2008). For the Chinese version of the ZKPQ-III-R, Wu et al. (2000) reported that internal consistency reliability (Cronbach’s α) ranged from 0.61 to 0.81 in general Chinese samples. Principal component analysis detected 16 factors with eigenvalues greater than 1.5, and the first five of 16 factors accounted for 21.0% of the variance. Wang et al. (2002) reported that internal consistency reliability (Cronbach’s α) ranged from 0.52 to 0.81 in Chinese college students without siblings. In another study, Wang et al. (2004) reported Cronbach’s α coefficients from 0.60 to 0.81. Chai et al. (2013) reported internal consistency reliability from 0.61 to 0.81 in Chinese middle school students. The ZKPQ-III-R has also been validated as a powerful tool for personality assessment in adolescents (Aluja et al., 2002).

To our knowledge, four short versions of the ZKPQ-III-R have been developed. Zuckerman (2002) developed a 35-item short form (ZKPQ-S) with seven items per subscale. Several items were excluded because of high correlation with other selected items (Zuckerman, 2002). These short scales had Cronbach’s α coefficients between 0.62 and 0.79. Aluja et al. (2003) proposed a 69-item short version of the ZKPQ based on exploratory and confirmatory factor analysis (CFA). Internal consistency was similar to the 89-item version, varying between 0.71 and 0.81. Aluja et al. (2006) proposed a third shortened form, the ZKPQ-50-CC, which consisted of 50 items that were selected based on their factor loadings and cross-language stability. Rahim et al. (2013) subsequently proposed a fourth shortened form, the ZKPQ-40-CC. This reduced version was based on the original ZKPQ-50-CC using different procedures for item analysis that were performed simultaneously in Malaysia samples. The results showed that the ZKPQ-40-CC had good Cronbach’s α coefficients, ranging from 0.76 to 0.84 for each domain. The decision regarding how many items to include in these short versions has varied, based on individual researchers’ needs. In most cases, however, a data-driven procedure is used, based on statistical criteria. These short versions can save administration time, with only negligible reductions of reliability (Rahim et al., 2013). Completing large questionnaires can cause boredom in subjects, which can produce transient measurement errors, in which subjects respond carelessly because of frustration with the length of the assessment. Given these practical concerns, researchers often create shortened versions of long assessment instruments (Stanton et al., 2002). Therefore, a Chinese short version of the ZKPQ was developed.

The ZKPQ-III-R has been widely used in general and clinical populations across various ages and cultures. Previous studies have indicated that these dimensions have good validity with regard to some constructs in the field of psychiatry, such as depression and other psychiatric problems. Wang et al. (2002) found that depression scores were positively correlated with N-Anx and Agg-Host. Chai et al. (2013) reported that Adolescent Health Related Risky Behavior Inventory (AHRBI) scores were positively correlated with ImpSS scores, and Barratt Impulsiveness Scale (BIS) scores were positively correlated with ImpSS, N-Anx, and Agg-Host scores. Giannoni-Pastor et al. (2014) noted that high N-Anx and Agg-Host were related to higher depression scores compared with low N-Anx and Agg-Host groups over 6 months of follow-up.

The present study examined the structure and psychometric properties of the Chinese short version of the ZKPQ-III-R in Chinese adolescents and young adults in an attempt to provide a well-adapted instrument for personality assessment in the Chinese sociocultural context based on the alternative Five-Factor model that was proposed by Zuckerman (2002). The present study also examined the validity of the short version of the ZKPQ-III-R by correlating ZKPQ-III-R dimensions with depression, subjective health complaints (SHC), life satisfaction, Big Five personality traits, and substance use risk profiles in Chinese adolescents and young adults.

## Materials and Methods

### Participants and Procedure

The survey was conducted between March and April 2016. The study included 1,019 adolescents and young adults who were enrolled in two senior middle schools and two universities in Anhui and Beijing, China. Two classes were randomly selected at each grade level. For the present study, data were obtained via the Internet. On-line versions of personality questionnaires have good equivalence and similar psychometric properties to traditional paper and pencil forms (Gosling et al., 2004; Aluja et al., 2007). All of the participants did not suffer from any psychiatric disorder currently and free from alcohol or drug use at least 72 h prior to participating in the study. Written consent was obtained from each participant after a full explanation of the study procedure. Parents/guardians of participants under 18 years old were informed, and their consent was obtained. The study was reviewed and approved by the Institutional Review Board of Human Research Ethics Committee for Non-Clinical Faculties of Collaborative Innovation Center of Assessment toward Basic Education Quality (CICA-BEQ) at Beijing Normal University.

A total sample of 1,019 healthy Chinese adolescents and young adults (51.70% females) were recruited for the study. Their mean age was 19.71 ± 1.41 years old (range, 14–25 years). The mean age for the females was 19.67 ± 1.46 years old (range, 14–24 years). The mean age for the males was 19.77 ± 1.35 years old (range, 14–25 years).

### Instruments

#### Zuckerman–Kuhlman Personality Questionnaire

The ZKPQ consists of 89 true-false items. The test measures five subscales: ImpSS (19 items), N-Anx (19 items), Agg-Host (17 items), Act (17 items), and Sy (17 items). In this questionnaire, 10 items from another scale about dissimulation (infrequency or lie) were randomly inserted into the test. The infrequency scale was used as an indicator of test validity for individuals (Zuckerman, 2002). For the original 89-item Chinese version of the ZKPQ-III-R, Wu et al. (2000) reported internal consistency reliability estimates (Cronbach’s α) that ranged from 0.61 to 0.81 for the five scales in a general Chinese sample. In the present study, the original 89-item version of the ZKPQ-III-R had virtually identical internal consistency reliability estimates (Cronbach’s α) that ranged from 0.61 to 0.82.

#### Beck Depression Inventory

Depressive symptoms were measured using the 13-item Beck Depression Inventory (BDI) (Beck and Beck, 1972). The 13-item form was developed for screening purposes and has been shown to be a valid measure for detecting depression. Each of the 13 items measured the presence and severity of depressive symptoms using self-ratings from 0 to 3. This is a rapid screening tool that consists of 13 questions with the following scoring scheme: no depression (0–4), mild depression (5–7), moderate depression (8–15), and severe depression (16 or above). The BDI has been validated for use in Chinese population (Wu et al., 2010; Zhang et al., 2015). In the present study, the BDI had internal consistency reliability of 0.88.

#### Satisfaction with Life Scale

The Satisfaction with Life Scale (SWLS) was developed by Diener et al. (1985). The SWLS consists of five items. Participants indicated the extent to which they agreed with each item on a 7-point scale, from strongly agree to strongly disagree. The scores were reversed and given in a range from 5 to 35, from poorly satisfied to highly satisfied, with 20 as the midpoint. The Chinese version of the SWLS has been shown to have good reliability and validity, with a Cronbach’s α coefficient of 0.78 and split-half reliability of 0.70 (Wang et al., 2009; Ma and Chan, 2015). In the present study, the SWLS had internal consistency reliability of 0.89.

#### Subjective Health Complaints

Subjective health complaints were measured using the Health Behavior in School-aged Children Symptom Checklist (HBSC-SCL) (Haugland and Wold, 2001), including headaches, abdominal pain, backache, feeling low, irritability, nervousness, sleeping difficulties, and dizziness. The participants reported on a 5-point scale whether each symptom was experienced most days, more than once per week, about once per week, about once every month, or seldom or never. The SHC index is calculated using only seven of the eight items: the item “sleeping difficulties” is not used because it shows differential item functioning across countries. Values range from 1 to 35. Higher values indicate fewer and less severe health complaints. The SHC checklist was previously found to be sensitive to the presence of psychosomatic disorders and psychological distress (Haugland et al., 2001). In the present study, the SHC had internal consistency reliability of 0.86.

#### Mini International Personality Item Pool-Five Factor Model Personality Scale

The Mini-IPIP scale measures the Big Five personality traits of neuroticism (N), extraversion (E), agreeableness (A), conscientiousness (C), and Intellect/Imagination (I). It consists of 20 items, with four items for each trait (Donnellan et al., 2006). Each item was a statement that described a behavior (e.g., “have frequent mood swings”), and the participants were asked to indicate the extent to which each statement generally described themselves using a 5-point Likert-type scale, with anchors of 1 (strongly disagree) and 5 (strongly agree). The Chinese version of the Mini-IPIP has been shown to have good test-retest reliability and convergent, discriminant, and criterion-related validities (Zheng et al., 2008; Li et al., 2012). In the present study, the Mini-IPIP had Cronbach’s α coefficients of 0.76 for N, 0.68 for E, 0.72 for A, 0.71 for C, and 0.84 for I.

#### Substance Use Risk Profile Scale

The original English version of the Substance Use Risk Profile Scale (SURPS) is a 28-item self-report inventory (Woicik et al., 2009). We translated the SURPS into Chinese and ensured fidelity through back translation and by addressing any problematic items. Discrepancies were discussed until agreement was reached. This version was then refined, paying special attention to the use of frequent and well-known words and using correct and easy grammar to ensure that the items were well understood by respondents for every level of education. The translation of the SURPS was approved by Woicik, the scale’s original author. The SURPS distinguishes four personality dimensions: anxiety sensitivity (AS), hopelessness (H), sensation seeking (SS), and impulsivity (IMP). Each dimension was assessed using six to eight items, and each item could be answered on a 4-point scale, ranging from 1 (strongly agree) to 4 (strongly disagree). In the present study, the SURPS had Cronbach’s α coefficients of 0.79 for AS, 0.72 for H, 0.66 for SS, and 0.77 for IMP.

### Statistical Analyses

Responses to all of the items were subjected to principal component factor analysis. The statistical analyses were performed using SPSS 22.0 (Chicago, IL, USA) and MPLUS 7 (Los Angeles, CA, USA). To obtain a shortened version, we used a series of exploratory and confirmatory factor analyses. Some items were removed after each analysis based on the criteria that are described below. Cronbach’s α was calculated for each dimensions of the ZKPQ-III-R. The analysis also included factor analysis with promax rotation and calculations of Pearson correlation coefficients between the subscale scores. The factor analysis was computed separately for the ZKPQ five-factor model together with eigenvalues ≥ 1. Items were deleted based on the following criteria: (a) items in each pair with lower loadings (i.e., loading ≤ 0.30 on the remaining factors), (b) items that correlated with more than one item, and (c) items with modification indices (MIs) ≥ 50 after CFA.

We used the following indices to assess the model fit: χ^2^/df, root mean square error of approximation (RMSEA), confidence interval (CI), weighted root mean square residual (WRMR), comparative fit index (CFI), and Tucker-Lewis index (TLI). According to generally accepted criteria, a good fit would be indicated by CFI > 0.95 and TLI > 0.95, and an acceptable fit would be indicated by CFI > 0.90 and TLI > 0.90. RMSEA values ≤ 0.05 would be considered good. RMSEA values between 0.05 and 0.08 would be considered adequate, and RMSEA values between 0.08 and 0.10 would be considered mediocre. Values of *p* < 0.05 were considered statistically significant (Schermelleh-Engel et al., 2003). Cohen’s *d* was used as an index of effect size. Cohen defined an effect size of *d* = 0.20 as small, *d* = 0.50 as medium, and *d* = 0.80 as large (Cohen, 1988). Internal consistencies were assessed by Cronbach’s α coefficient. Concurrent validity of the Chinese short 46-items version ZKPQ-III-R was examined by correlating five dimensions of the ZKPQ-III-R with measures of depression (BDI), life satisfaction (SWLS), SHC, Big Five personality traits (Mini-IPIP), and the SURPS.

## Results

### Exploratory Factor Analysis

A principal component analysis with Varimax rotation was performed for the original 89-item ZKPQ-III-R, excluding the 10 Inf subscale items. Five factors were extracted, which explained 27.27% of the total variance. Most of the items were encapsulated in their respective factors according to the original distribution of the questionnaire. The items that loaded in different factors were the following: 30 (ImpSS) loading on N-Anx, 16 and 57 (ImpSS) loading on Agg-Host, 48 and 49 loading on Act, 22 and 63 (ImpSS) loading on Sy, 99 and 23 (N-Anx) loading on Act, 43 (N-Anx) loading on Sy, 95, 50, 84, 89, 1, 19, 45, and 14 (Agg-Host) loading on ImpSS, 44 (Agg-Host) loading on Act, 29, 39, 75, 34, and 45 (Act) loading on ImpSS, 31 and 36 (Act) loading on Agg-Host, 78, 48, 82, 98, 9, 53, and 27 (Act) loading on Sy, 38 (Sy) loading on Act, and 75 (Sy) loading on ImpSS. The following items had loading < 0.30: 6, 50, 29, 39, 75, 84, 34, 89, 1, 19, 45, 14, 85, 2, 36, 47, 16, 86, 57, 31, 62, 21, 94, 99, 44, 88, 49, 23, 38, 78, 48, 82, 98, 9, 53, 27, 92, and 58. All of these items were deleted, and a second factor analysis was performed with the 51 remaining items. Of these, 33.75% of the total variance was explained by extracting five factors. In this analysis, only item 43 had a loading < 0.30, so it was also deleted. Two additional items were deleted because of the following additional criteria (explained in the CFA section). A third factor analysis was performed with the 46 remaining items, and the five factors accounted for 34.22% of the total variance (see Supplementary Table 2).

### Confirmatory Factor Analysis

Beginning with the 50-item solution (excluding item 43), an orthogonal CFA was performed over the variance-covariance matrix using MPLUS 7. The maximum likelihood estimation method was used. To achieve model identification, regression coefficients of the error terms over the endogenous variables were fixed to 1. The CFA was performed to test the goodness-of-fit of the five-factor model. This procedure allowed the calculation of error variances among the items. The analysis of error variance showed that four item pairs were highly correlated, and the MIs were > 50. This indicated that there were probably very similar items in the content, so the subjects tended to answer in the same way. The item pairs were the following: 46–51 (MI = 156.63, *r* = 0.42); 24–55 (MI = 100.42, *r* = 0.51); 41–56 (MI = 67.67, *r* = 0.31); 61–71 (MI = 67.16, *r* = 0.40). The content analysis of these items revealed that they were somehow redundant, and deleting one item of each pair has been recommended for convenience (Byrne, 1993; Yadama and Drake, 1995). The item of each pair that had the lower loading was deleted (24, 51, 56, and 71). With these modifications, the questionnaire was reduced to 46 items.

In **Table 1**, the CFA models of the 89-item version, 50-item version, and 46-item version of the ZKPQ-III-R were compared with the original model with regard to (a) the CFA of the 89-item version, 50-item version, and 46-item version, (b) the secondary loadings of the 89-item version, 50-item version, and 46-item version, and (c) the correlated error terms of the 89-item version, 50-item version, and 46-item version. As shown in **Table 1**, the 46-item version comprised items with secondary loadings > 0.20 and error variances with MIs > 50 (33–54, 3–8, 5–33, 28–74, 41–95, 15–41, and 7–20 item pairs).

**Table 1:** Comparison of several ZKPQ-III-R fit indices for the estimated models.

CFA Model	χ^2^	df	χ^2^/df	CFI	TLI	RMSEA	90% CI	WRMR
89-item^a^	11246.20	3817	2.95	0.61	0.60	0.044	0.043–0.045	2.176
89-SL^b^	10264.86	3803	2.70	0.66	0.65	0.041	0.040–0.042	2.040
89-SL_r^c^	10011.16	3791	2.64	0.70	0.66	0.040	0.039–0.041	2.016
50-item^a^	2889.41	1165	2.48	0.81	0.80	0.043	0.041–0.045	1.650
50-SL^b^	2581.75	1159	2.23	0.84	0.83	0.039	0.037–0.041	1.531
50-SL_r^c^	2661.12	1156	2.30	0.83	0.82	0.040	0.038–0.042	1.564
46-item^a^	3145.89	1070	2.94	0.82	0.81	0.044	0.042–0.045	1.765
46-SL^b^	2587.11	1061	2.44	0.87	0.86	0.038	0.036–0.039	1.560
46-SL_r^c^	2954.44	1063	2.78	0.84	0.83	0.042	0.040–0.044	1.697

*n = 1019. All χ^2^ values were significant (*p* < 0.001). CFA, confirmatory factor analysis; CFI, comparative fit index; TLI, Tucker-Lewis index; CI, confidence interval; RMSEA, root mean square error of approximation; WRMR, weighted root mean square residual; χ^2^/df, the associated *p*-values were always < 0.001.*

The goodness-of-fit indices of the simple structure of the ZKPQ-III-R for the 89-item version were generally very low, with the exception of the RMSEA and SRMR. The 89-item version that was derived from the first CFA was nearly identical to the original version. The 50-item version that was obtained from the second CFA yielded fit indices that were slightly better than the previous model. The 46-item version that was derived from the third CFA was also improved compared with the previous models. Both specifications of this model yielded the most acceptable indices, especially χ^2^/df < 3 and RMSEA < 0.05 (Byrne, 1993; Yadama and Drake, 1995).

Following the same CFA procedure, **Table 2** shows the goodness-of-fit indicators that were used to separately analyze each of the five independent factors of the 46-item model. In this case, the fit indices were excellent for the ImpSS and Sy subscales and good for the N-Anx, Agg-Host, and Act subscales. These results showed that five factors in the 46-item model were consistent, which was further confirmed when the standardized regression coefficients were considered.

**Table 2:** Fit indices of the independent factors of the 46-item version of the ZKPQ-III-R.

CFA Model	χ^2^	df	χ^2^/df	CFI	TLI	RMSEA	90% CI	WRMR
ImpSS	26.73	9	2.97	0.97	0.96	0.044	0.025–0.064	0.948
N-Anx	519.35	104	4.99	0.93	0.92	0.063	0.057–0.068	1.714
Agg-Host	142.57	27	5.28	0.93	0.91	0.065	0.055–0.075	1.539
Act	260.37	35	7.44	0.91	0.89	0.079	0.071–0.089	1.882
Sy	55.67	14	3.98	0.97	0.95	0.054	0.040–0.069	1.199

*n = 1019. All χ^2^ values were significant (*p* < 0.001). CFA, confirmatory factor analysis; CFI, comparative fit index; TLI, Tucker-Lewis index; CI, confidence interval; RMSEA, root mean square error of approximation; WRMR, weighted root mean square residual; ImpSS, Impulsive Sensation Seeking; N-Anx, Neuroticism-Anxiety; Agg-Host, Aggression-Hostility; Act, Activity; and Sy, Sociability.*

### Descriptive Characteristics

Descriptive characteristics summarizes the means, standard deviations, kurtosis, skewness, *t*-tests, and coefficients for the 89-item original version of the ZKPQ-III-R, including the Inf scale, and the 46-item shortened version. The criteria for selecting the 46-item model are described below.

In the 89-item version, the means were very similar to those reported by Zuckerman et al. (1993). Men had higher scores than women on ImpSS (9.57 vs. 8.97, *p* < 0.01, *d* = 0.16), Agg-Host (6.85 vs. 6.38, *p* < 0.01, *d* = 0.17), Act (8.14 vs. 7.01, *p* < 0.01, *d* = 0.37), Sy (8.31 vs. 7.54, *p* < 0.01, *d* = 0.25), and Inf (3.49 vs. 2.04, *p* < 0.01, *d* = 0.65). Women had higher scores than men on N-Anx (10.67 vs. 9.64, *p* < 0.01, *d* = 0.23). The α coefficients were also similar to those reported by Zuckerman et al. (1993), Wu et al. (2000), and Wang et al. (2002), ranging from 0.58 to 0.82 (see Supplementary Table 1).

Descriptive data for the 46-item shortened version of the ZKPQ-III-R had Cronbach’s α coefficients of 0.63 for ImpSS, 0.84 for N-Anx, 0.70 for Agg-Host, 0.73 for Act, and 0.63 for Sy, indicating that the scales of the shortened version maintained internal consistency that was similar to the 89-item original version and 89-item Chinese version. The ratio of kurtosis can be used as a test of normality. According to Curran et al. (1996), for univariate normality, skewness absolute values of 0–2 and kurtosis absolute values of 0–7 can be considered sufficient normality. The kurtosis and skewness values indicated that all of the scales had a normal and symmetrical distribution for both the 89- and 46-item versions of the ZKPQ-III-R.

### Correlational Analysis of ZKPQ-III-R with BDI, SWLS, SHC, SURPS, and Mini-IPIP

**Table 3** shows two correlation matrices among the five ZKPQ-III-R scales of the original 89-item version and 46-item shortened version. ImpSS yielded moderate correlations with N-Anx, Agg-Host, Act, and Sy (0.43, 0.28, 0.25, and 0.08, respectively; *p* < 0.05). N-Anx was related to Agg-Host, Act, and Sy (0.25, 0.10, and -0.11; *p* < 0.05). Agg-Host was also correlated with Act (0.08; *p* < 0.05), and Act was correlated with Sy (0.31; *p* < 0.05). Inter-correlations of the original 89-item version and 46-item shorted version of the ZKPQ-III-R followed a very similar pattern throughout every scale.

**Table 3:** Inter-correlations among the 89-item and 46-item version of the ZKPQ-III-R and Pearson correlation coefficients of ZKPQ-III-R domain scores and BDI, SWLS, SHC, SURPS, and Mini-IPIP scores.

*n* = 1019	ZKPQ-III-R original 89-item					ZKPQ-III-R 46-item				
	ImpSS	N-Anx	Agg-Host	Act	Sy	ImpSS	N-Anx	Agg-Host	Act	Sy
ImpSS	-					-				
N-Anx	**0.43^∗∗^**	-				**0.38^∗∗^**	-			
Agg-Host	0.28^∗∗^	0.25^∗∗^	-			0.22^∗∗^	0.44^∗∗^	-		
Act	0.25^∗∗^	0.10^∗∗^	0.08^∗^	-		**0.31^∗∗^**	0.24^∗∗^	**0.31^∗∗^**	-	
Sy	0.08^∗^	-0.11^∗∗^	0.02	**0.31^∗∗^**	-	-0.18^∗^	-**0.31^∗∗^**	-0.27^∗∗^	-0.17^∗∗^	-
BDI	0.11^∗∗^	**0.36^∗∗^**	0.26^∗∗^	-0.10^∗∗^	-0.18^∗∗^	-0.04	**0.33 ^∗∗^**	0.24^∗∗^	-0.07^∗^	-0.17^∗∗^
SWLS	-0.08	-0.15^∗∗^	0.06	0.10^∗^	-0.09^∗^	-0.16^∗∗^	-0.16^∗∗^	-0.04	0.14^∗∗^	0.09^∗^
SHC	-0.15^∗∗^	-**0.34^∗∗^**	-0.22^∗∗^	0.02	0.11^∗∗^	-0.07^∗^	-**0.35^∗∗^**	-0.22^∗∗^	-0.01	0.13^∗∗^
SURPS-H	0.04	**0.35^∗∗^**	0.15^∗∗^	-0.17^∗∗^	-0.29^∗∗^	-0.05	**0.31^∗∗^**	0.10	-0.21^∗∗^	-0.21^∗∗^
SURPS-AS	0.13^∗^	**0.44^∗∗^**	0.11^∗^	0.01	-0.10	0.07	**0.42^∗∗^**	0.13^∗^	0.03	-0.10
SURPS-IMP	0.26^∗∗^	0.29^∗∗^	0.28^∗∗^	0.09	-0.14^∗∗^	0.06	0.26^∗∗^	**0.30^∗∗^**	0.07	-0.17^∗∗^
SURPS-SS	**0.31^∗∗^**	0.06	0.09	0.22^∗∗^	-0.01	0.25^∗∗^	0.05	0.12^∗^	0.25^∗∗^	-0.16^∗∗^
Mini-IPIP-E	0.09	-0.13^∗^	0.03	0.24^∗∗^	**0.53^∗∗^**	0.13^∗^	-0.12^∗^	0.07	0.20^∗∗^	**0.39^∗∗^**
Mini-IPIP-A	-0.07	-0.08	-0.23^∗∗^	0.12^∗^	**0.30^∗∗^**	0.12^∗^	-0.08	-0.19^∗∗^	0.09	0.27^∗∗^
Mini-IPIP-C	-0.19^∗∗^	-0.10	-0.21^∗∗^	0.17^∗∗^	-0.02	-0.01	-0.09	-0.20^∗∗^	0.14^∗∗^	0.00
Mini-IPIP-N	0.25^∗∗^	**0.55^∗∗^**	**0.34^∗∗^**	0.07	-0.14^∗∗^	0.10	**0.53^∗∗^**	**0.36^∗∗^**	0.08	-0.14^∗∗^
Mini-IPIP-I	0.18^∗∗^	0.00	-0.10	0.07	0.07	0.26^∗∗^	0.02	-0.03	0.10	0.01

*Correlations > 0.30 are shown in bold. ImpSS, Impulsive Sensation Seeking; N-Anx, Neuroticism-Anxiety; Agg-Host, Aggression-Hostility; Act, Activity; Sy, Sociability; BDI, Beck Depression Inventory-13 item; SWLS, Satisfaction with Life Scale; SHC, Subjective Health Complaints; SURPS, Substance Use Risk Profile Scale; H, Hopelessness; AS, Anxiety Sensitivity; IMP, Impulsivity; SS, Sensation Seeking; Mini-IPIP, Mini International Personality Item Pool; E, Extraversion; A, Agreeableness; C, Conscientiousness; N, Neuroticism; I, Intellect/Imagination. ^∗^*p* < 0.05, ^∗∗^*p* < 0.01.*

**Table 3** also shows the correlation matrix between the ZKPQ-III-R and BDI, SWLS, SHC, SURPS, and Mini-IPIP, including the original 89-item version and 46-item shortened version. Both versions of the ZKPQ-III-R exhibited very similar values. The BDI had moderate correlations with N-Anx (0.36 and 0.33) and Agg-Host (0.26 and 0.24) and somewhat lower negative correlations with Act (-0.10 and -0.07) and Sy (-0.18 and -0.17). The SWLS was negatively correlated with N-Anx (-0.15 and -0.16) and positively correlated with Act (0.10 and 0.14). The SHC was negatively correlated with ImpSS (-0.15 and -0.07), N-Anx (-0.34 and -0.35), and Agg-Host (-0.22 and -0.22) and positively correlated with Sy (0.11 and 0.13). These findings that showed that all of the correlations were significant at *p* < 0.05 were consistent with previous studies (Zuckerman et al., 1993).

The SURPS-H was correlated with N-Anx (0.35 and 0.31). The SURPS-AS was correlated with N-Anx (0.44 and 0.42). The SURPS-IMP was correlated with Agg-Host (0.29 and 0.30). The SURPS-SS was correlated with ImpSS (0.31 and 0.25). The SURPS scales were essentially related to ImpSS and also related to Agg-Hos, Act, and Sy, although to a lesser extent. Furthermore, the Mini-IPIP-E was related to Sy (0.53 and 0.39). The Mini-IPIP-A was related to Sy (0.30 and 0.27). The Mini-IPIP-N was related to N-Anx (0.55 and 0.53) and Agg-Host (0.34 and 0.36). All of these correlations were significant at *p* < 0.01. These results are strongly similar to the factor structure that was obtained in studies in which the ZKPQ-III-R, NEO-PI-R, and EPQ-R were analyzed together (Aluja et al., 2003).

Finally, the relationships between the SURPS and Mini-IPIP facets produced additional evidence of the construct validity of the ZKPQ-III-R. Note that every ZKPQ-III-R scale in both the 89- and 46-item versions had high correlations with their theoretically corresponding SURPS facets: SURPS-H (Hopelessness) and SURPS-AS (Anxiety Sensitivity) for N-Anx, SURPS-IMP (Impulsivity) for ImpSS, and SURPS-SS (Sensation Seeking) for ImpSS. Every ZKPQ-III-R scale in both the 89- and 46-item versions had high correlations with their theoretically corresponding Mini-IPIP facets: Mini-IPIP-E (Extraversion) for Sy, Mini-IPIP-A (Agreeableness) for Sy, and Mini-IPIP-N (Neuroticism) for N-Anx and Agg-Host.

## Discussion

The main objective of the present study was to examine the psychometric properties and factor structure of the ZKPQIII-R and validate it by correlating its main dimensions with the BDI, SWLS, SHC, SURPS, and Mini-IPIP in Chinese adolescents and young adults (*n* = 1019). The structure of the Chinese version of the ZKPQ-III-R, ascertained by EFA procedures, indicated that the 89-item Chinese version is similar to the original 89-item English version, but 35 items loaded on different factors. Thirty-eight items were deleted for subsequent analysis, given that the obtained factor loadings were <0.30. Furthermore, four item pairs had unexpectedly high correlations, which might have had a negative impact on fit indices when testing the model using CFA. This also provided support for deleting one item in each pair so that the redundant information could be eliminated.

Several authors have used CFA to study the factor structure of personality questionnaires (Rossier et al., 2008; Cooper et al., 2010; Wielkiewicz, 2015). In the French version of ZKPQ and 69 items of the ZKPQ-R, there are a large number of facets yielding loadings higher than 0.20 on other factor, and it possibly leads to unacceptable fit to data when we perform CFA over simple structure models (Aluja et al., 2003; Rossier et al., 2008). It has been suggested that it is necessary to include secondary loadings in models that are generated by CFA to improve fit indices, although on some occasions it may be necessary to utilize the complete structure to obtain an acceptable fit to the data (Mccrae et al., 1996).

In the present study, we used CFA to compare the fits of the items to their respective using EFA generated factors. The fit indices of nine ZKPQ-III-R models that were determined using CFA was compared to the 89-, 51-, and 46-item versions. The modified 89-item Chinese model and 50-item model were very similar although rather poor. The simple structure of the 46-item model had low indices, although these indices were higher than those that were reported elsewhere with several personality structural models that were derived through CFA (Aluja et al., 2003; Rossier et al., 2008). In the 46-item CFA structure, the secondary loadings were low, but the fit improved when correlated error terms were added. When the five factors of the 46-item version of the ZKPQ-III-R were analyzed independently using CFA, the fit indices were adequate, supporting the construct validity of the remaining scales.

In the present study, the 46-item shortened version revealed many intercorrelations among the five dimensions that were considerably larger compared with the 89-item original version. The correlations between the N-Anx and Act dimensions and the other dimensions (i.e., ImpSS, Agg-Host, and Sy) were strengthened. Nonetheless, the intercorrelations among the five dimensions of the 46-version were similar to the 89-item original version.

Convergent validity of the ZKPQ-III-R has been studied using correlational analyses with the BDI, SWLS, SHC, SURPS, and Mini-IPIP. High convergence has been reported between the ZKPQ-III-R and its equivalent scales in the other five instruments, corroborating the outcomes that were found by Zuckerman et al. (1993); Aluja et al. (2003), and Rossier et al. (2008). Correlation magnitudes were very similar to the 89-item original version and 46-item shortened version. Internal consistency coefficients were acceptable and similar to those in Zuckerman et al. (1993) and other studies with Chinese samples. For the Chinese version of the ZKPQ-III-R, Wu et al. (2000) reported the following internal consistency reliabilities (Cronbach’s α) in general Chinese samples: 0.81 for N-Anx, 0.68 for ImpSS, 0.63 for Sy, 0.62 for Agg-Host, and 0.61 for Act. Wang et al. (2002) reported the following internal consistency reliabilities (Cronbach’s α) in Chinese college students without siblings: 0.81 for N-Anx, 0.74 for ImpSS, 0.59 for Sy, 0.52 for Agg-Host, and 0.64 for Act. Wang et al. (2004) reported Cronbach’s α coefficients of 0.81 for N-Anx, 0.71 for ImpSS, 0.68 for Act, 0.64 for Sy, and 0.60 for Agg-Host. Chai et al. (2013) reported the following internal consistency reliabilities in Chinese middle school students: 0.81 for N-Anx, 0.68 for ImpSS, 0.63 for Sy, 0.62 for Agg-Host, and 0.61 for Act. The α coefficient of the 46-item version was also adequate, and the deletion of items did not appear to affect the questionnaire’s reliability.

The ZKPQ-III-R is widely used in cross-cultural fields. It has been translated and revised in many languages, including French, Spanish, Chinese, and Malaysian, among others. The validity and reliability of the 89-item Chinese adaptation of the ZKPQ-III-R was shown to be similar to the 89-item original version, although some items in the Chinese version loaded on different factors.

The present results showed that the 46-item shortened version had higher validity than and attained similar reliability coefficients as the 89-item original version and 89-item Chinese version. The present study also found that the 46-item shortened version of the ZKPQ-III-R had adequate internal consistency reliability for all five dimensions. The concurrent validity of the 46-item shortened version of the ZKPQ-III-R was supported by significant correlations with depression, life satisfaction, and SHC. The 46-item shortened version of the ZKPQ-III-R had better fit, similar reliability coefficients, and slightly better construct and convergent validity than the 89-item version. The present study supports the hypothesis that the alternative five-factor model of the ZKPQ has good reliability with regard to constructs and internal reliability in Chinese adolescents and young adults. The 46-item shortened version of the ZKPQ-III-R may be a useful tool for researchers who need a short assessment of the alternative five-factor.

The present study has several limitations. First, no clear criteria for factor selection were articulated for the EFA, and eigenvalues > 1 appeared to be the sole basis for retaining factors. Second, deleting items based only on a single principal components analysis (PCA) with Varimax rotation may be controversial. Third, the sample consisted of adolescents and young adults only from Anhui and Beijing. Future studies should recruit more participants from other areas in China. Fourth, the present study was data-driven, without considering the relative equilibrium of the number of items in each dimension. This should be addressed in future studies.

## Author Contributions

DW: Guarantor of integrity of entire study, study concepts, study design, literature research, data acquisition, data analysis/interpretation, statistical analysis, manuscript preparation, and manuscript final version approval. MH: literature research, data acquisition, data analysis/interpretation, statistical analysis, manuscript preparation, manuscript final version approval. CZ: literature research, data analysis/interpretation, statistical analysis, manuscript definition of intellectual content, manuscript editing, manuscript revision, and manuscript final version approval. ZL: literature research, guarantor of integrity of entire study, study concepts, study design, data analysis/interpretation, statistical analysis, manuscript definition of intellectual content, manuscript editing, manuscript revision, and manuscript final version approval.

## Conflict of Interest Statement

The authors declare that the research was conducted in the absence of any commercial or financial relationships that could be construed as a potential conflict of interest.
