# Supplementary material for: Psychometric Properties of the Chinese Shortened Version of the Zuckerman–Kuhlman Personality Questionnaire in a Sample of Adolescents and Young Adults
Source: Front Psychol. 2017 Mar 7;8:349. doi: 10.3389/fpsyg.2017.00349 (PMC5339253; doi:10.3389/fpsyg.2017.00349)
Supplement: Supplementary file 1 [file Table_1.DOC]

Supplementary material:

Some studies also indicated that there might be gender difference in ZKPQ-III-R scores. Rossier et al. (2008) reported that women had significantly higher N-Anx scores and significantly lower ImpSS and Act scores. A facet-level analysis showed that women had significantly lower scores for Sensation Seeking, Work Effort, and Parties and Friends, and higher scores for Intolerance of social Isolation than men. Wang et al. (2004) reported that men had significantly higher Agg-Host scores. Chai et al. (2013) reported that girls had significantly higher N-Anx scores than boys.

**Table 1.** Means, standard deviations (SD), and gender differences on ZKPQ-III-R scales and subscales. The table also shows the internal consistency reliability (α) of the ZKPQ from an American study^a^ (Zuckerman et al., 1993), Chinese general sample study^b^ (Wu et al., 2000), Chinese general university student sample study^c^ (Wang et al., 2002), and the present study.^d^

| Subscale | Item | Male (*n* = 492) | | |  | Female (*n* = 527) | | |  | All (*n* = 1019) | | | |  | Internal reliability | | | |
| --- | --- | --- | --- | --- | --- | --- | --- | --- | --- | --- | --- | --- | --- | --- | --- | --- | --- | --- |
|  |  | Mean | SD | α |  | Mean | SD | α |  | *t*-test | Cohen’s *d* | Kurtosis | Skewness |  | a | b | c | d |
| ZKPQ-III-R original 89-item version | | | | |  |  |  |  |  |  |  |  |  |  |  |  |  |  |
| ImpSS | 19 | 9.57 | 3.83 | 0.74 |  | 8.97 | 3.53 | 0.71 |  | 2.60* | 0.16 | 0.02 | -0.51 |  | 0.82 | 0.68 | 0.74 | 0.72 |
| N-Anx | 19 | 9.64 | 4.48 | 0.82 |  | 10.67 | 4.23 | 0.81 |  | -3.74** | -0.23 | -0.12 | -0.82 |  | 0.86 | 0.81 | 0.81 | 0.82 |
| Agg-Host | 17 | 6.85 | 2.94 | 0.58 |  | 6.38 | 2.81 | 0.58 |  | 2.65** | 0.17 | 0.14 | -0.53 |  | 0.76 | 0.62 | 0.52 | 0.58 |
| Act | 17 | 8.14 | 3.08 | 0.60 |  | 7.01 | 3.06 | 0.63 |  | 5.89** | 0.37 | 0.02 | -0.56 |  | 0.72 | 0.61 | 0.64 | 0.63 |
| Sy | 17 | 8.31 | 3.08 | 0.61 |  | 7.54 | 2.97 | 0.61 |  | 4.02** | 0.25 | -0.24 | -0.15 |  | 0.81 | 0.63 | 0.59 | 0.61 |
| Inf | 10 | 3.49 | 2.53 | 0.73 |  | 2.04 | 1.89 | 0.64 |  | 10.36** | 0.65 | 1.11 | 0.92 |  | — | — | — | 0.73 |
| ZKPQ-III-R 46-item version | | | | |  |  |  |  |  |  |  |  |  |  |  |  |  |  |
| ImpSS | 6 | 3.65 | 1.77 | 0.67 |  | 3.78 | 1.60 | 0.59 |  | -1.21 | — | -0.51 | -0.59 |  | — | — | — | 0.63 |
| N-Anx | 14 | 7.76 | 4.09 | 0.85 |  | 8.68 | 3.74 | 0.83 |  | -3.76** | -0.24 | -0.18 | -0.73 |  | — | — | — | 0.84 |
| Agg-Host | 9 | 3.45 | 2.42 | 0.74 |  | 3.07 | 2.10 | 0.66 |  | 2.69** | 0.17 | 0.50 | -0.40 |  | — | — | — | 0.70 |
| Act | 10 | 4.47 | 2.77 | 0.76 |  | 3.64 | 2.41 | 0.68 |  | 6.77** | 0.43 | 0.35 | -0.73 |  | — | — | — | 0.73 |
| Sy | 7 | 3.69 | 1.85 | 0.60 |  | 4.02 | 1.90 | 0.65 |  | -2.83* | -0.18 | -0.12 | -0.96 |  | — | — | — | 0.63 |

Impulsive Sensation Seeking (ImpSS), Neuroticism-Anxiety (N-Anx), Aggression-Hostility (Agg-Host), Activity (Act), Sociability (Sy), and Infrequency (Inf).

ZKPQ-III-R 46-item version: ImpSS = 55, 60, 65, 70, 79, 95 (true); N-Anx = 7, 15, 20, 25, 30, 35, 41, 46, 61, 66, 76, 80, 90, 96 (true); Agg-Host = 3, 8, 11, 42, 67, 72, 77, 91, 97 (true); Act = 5, 13, 18, 28, 33, 54, 59, 64, 74, 83 (true); Sy = 22 (true); 12, 17, 37, 63, 68, 87 (false). **p* < 0.05, ***p* < 0.01.

**Table 2.** Exploratory factor analysis of three ZKPQ-III-R structures: 89, 50, and 48 items.

| Item | Principal components with varimax rotation of Chinese ZKPQ-III-R^a^ | | | | | | | | | | |  |  |  |  |  |  |
| --- | --- | --- | --- | --- | --- | --- | --- | --- | --- | --- | --- | --- | --- | --- | --- | --- | --- |
|  | 89-item | | | | |  | 50-item | | | | |  | 46-item | | | | |
|  | F-1 | F-2 | F-3 | F-4 | F-5 |  | F-1 | F-2 | F-3 | F-4 | F-5 |  | F-1 | F-2 | F-3 | F-4 | F-5 |
| 55 | 0.17 | -0.05 | **0.61** | 0.03 | -0.01 |  | 0.20 | -0.02 | **0.62** | -0.11 | -0.04 |  | 0.18 | -0.02 | **0.67** | -0.07 | -0.03 |
| 65 | 0.09 | 0.07 | **0.59** | 0.02 | -0.14 |  | 0.11 | 0.08 | **0.62** | -0.05 | -0.16 |  | 0.11 | 0.09 | **0.61** | -0.04 | -0.14 |
| 60 | 0.08 | 0.09 | **0.53** | 0.06 | 0.02 |  | 0.12 | 0.13 | **0.59** | -0.08 | -0.01 |  | 0.11 | 0.13 | **0.61** | -0.06 | 0.00 |
| 24 | -0.04 | 0.06 | **0.50** | 0.15 | 0.00 |  | -0.02 | 0.08 | **0.58** | 0.08 | 0.01 |  | ^c^— | — | — | — | — |
| 95 | 0.04 | 0.14 | **0.34** | 0.33 | -0.05 |  | 0.05 | 0.16 | **0.39** | 0.27 | -0.05 |  | 0.05 | 0.16 | **0.39** | 0.28 | -0.05 |
| 70 | 0.23 | 0.02 | **0.33** | 0.22 | -0.14 |  | 0.23 | 0.04 | **0.38** | 0.16 | -0.20 |  | 0.24 | 0.05 | **0.40** | 0.17 | -0.19 |
| 79 | 0.05 | 0.26 | **0.30** | 0.22 | 0.07 |  | 0.06 | 0.26 | **0.34** | 0.15 | 0.03 |  | 0.05 | 0.26 | **0.36** | 0.17 | 0.04 |
| 6 | -0.22 | -0.10 | **-0.29** | 0.16 | -0.08 |  | — | — | — | — | — |  | — | — | — | — | — |
| 50 | 0.16 | 0.13 | **0.27** | 0.31 | 0.02 |  | — | — | — | — | — |  | — | — | — | — | — |
| 29 | 0.05 | -0.31 | **-0.26** | 0.17 | 0.03 |  | — | — | — | — | — |  | — | — | — | — | — |
| 39 | -0.11 | 0.38 | **0.25** | 0.16 | -0.10 |  | — | — | — | — | — |  | — | — | — | — | — |
| 75 | 0.04 | 0.34 | **0.25** | 0.07 | -0.35 |  | — | — | — | — | — |  | — | — | — | — | — |
| 84 | 0.16 | 0.15 | **0.25** | 0.39 | -0.08 |  | — | — | — | — | — |  | — | — | — | — | — |
| 34 | -0.07 | 0.30 | **0.16** | 0.21 | -0.25 |  | — | — | — | — | — |  | — | — | — | — | — |
| 89 | 0.28 | 0.03 | **0.14** | 0.44 | 0.09 |  | — | — | — | — | — |  | — | — | — | — | — |
| 1 | 0.06 | 0.09 | **-0.11** | 0.40 | -0.19 |  | — | — | — | — | — |  | — | — | — | — | — |
| 19 | 0.08 | 0.02 | **0.06** | 0.40 | -0.20 |  | — | — | — | — | — |  | — | — | — | — | — |
| 45 | 0.11 | 0.30 | **0.02** | 0.36 | -0.24 |  | — | — | — | — | — |  | — | — | — | — | — |
| 14 | 0.19 | 0.07 | **0.01** | 0.46 | -0.16 |  | — | — | — | — | — |  | — | — | — | — | — |
|  |  |  |  |  |  |  |  |  |  |  |  |  |  |  |  |  |  |
| 61 | **0.63** | -0.14 | 0.12 | 0.08 | -0.04 |  | **0.65** | -0.13 | 0.07 | 0.03 | -0.04 |  | **0.65** | -0.13 | 0.07 | 0.04 | -0.04 |
| 46 | **0.61** | -0.05 | 0.19 | 0.07 | -0.13 |  | **0.62** | -0.03 | 0.18 | 0.03 | -0.15 |  | **0.64** | -0.02 | 0.17 | 0.02 | -0.14 |
| 41 | **0.60** | 0.07 | -0.05 | 0.12 | 0.00 |  | **0.60** | 0.05 | -0.07 | 0.16 | 0.01 |  | **0.61** | 0.06 | -0.08 | 0.15 | 0.02 |
| 66 | **0.59** | 0.04 | 0.24 | 0.06 | -0.03 |  | **0.60** | 0.05 | 0.23 | 0.03 | -0.05 |  | **0.61** | 0.06 | 0.22 | 0.03 | -0.03 |
| 51 | **0.55** | 0.14 | 0.01 | 0.09 | -0.04 |  | **0.59** | 0.15 | -0.02 | 0.03 | 0.01 |  | ^c^— | — | — | — | — |
| 96 | **0.55** | 0.08 | 0.02 | 0.08 | -0.01 |  | **0.56** | -0.09 | 0.32 | -0.04 | 0.08 |  | **0.56** | 0.05 | -0.01 | 0.09 | -0.01 |
| 71 | **0.55** | -0.07 | 0.36 | 0.00 | 0.07 |  | **0.56** | 0.20 | -0.06 | 0.07 | 0.03 |  | ^c^— | — | — | — | — |
| 56 | **0.53** | 0.19 | -0.03 | 0.09 | 0.00 |  | **0.55** | 0.05 | 0.00 | 0.10 | -0.01 |  | ^c^ — | — | — | — | — |
| 15 | **0.50** | 0.03 | -0.03 | 0.24 | -0.16 |  | **0.53** | 0.08 | 0.00 | 0.21 | -0.14 |  | **0.56** | 0.09 | -0.05 | 0.18 | -0.14 |
| 20 | **0.46** | -0.09 | 0.25 | 0.11 | -0.05 |  | **0.46** | -0.06 | 0.27 | 0.06 | -0.05 |  | **0.50** | -0.04 | 0.24 | 0.04 | -0.04 |
| 80 | **0.42** | 0.12 | -0.01 | 0.18 | -0.14 |  | **0.44** | 0.24 | 0.02 | 0.16 | -0.17 |  | **0.44** | 0.14 | 0.00 | 0.15 | -0.18 |
| 90 | **0.42** | 0.22 | 0.02 | 0.20 | -0.18 |  | **0.42** | 0.13 | 0.00 | 0.16 | -0.18 |  | **0.46** | 0.25 | 0.01 | 0.15 | -0.17 |
| 76 | **0.41** | 0.24 | -0.02 | 0.22 | 0.00 |  | **0.41** | -0.11 | 0.16 | 0.06 | -0.02 |  | **0.41** | 0.24 | -0.03 | 0.26 | 0.03 |
| 35 | **0.39** | -0.02 | 0.27 | 0.16 | -0.05 |  | **0.41** | 0.24 | -0.01 | 0.26 | 0.03 |  | **0.41** | 0.02 | 0.27 | 0.13 | -0.07 |
| 30 | **0.39** | -0.07 | 0.33 | 0.11 | -0.05 |  | **0.40** | 0.21 | 0.04 | 0.17 | -0.26 |  | **0.41** | -0.01 | 0.31 | 0.04 | -0.08 |
| 7 | **0.39** | -0.13 | 0.18 | 0.12 | -0.03 |  | **0.39** | 0.00 | 0.30 | 0.14 | -0.09 |  | **0.42** | -0.10 | 0.14 | 0.06 | -0.03 |
| 25 | **0.38** | 0.13 | 0.02 | 0.25 | -0.28 |  | **0.38** | -0.03 | 0.34 | 0.06 | -0.09 |  | **0.43** | 0.22 | 0.01 | 0.15 | -0.25 |
| 85 | **0.02** | -0.22 | -0.38 | 0.13 | 0.03 |  | — | — | — | — | — |  | — | — | — | — | — |
| 2 | **-0.01** | 0.09 | -0.21 | -0.17 | 0.12 |  | — | — | — | — | — |  | — | — | — | — | — |
|  |  |  |  |  |  |  |  |  |  |  |  |  |  |  |  |  |  |
| 72 | 0.25 | 0.10 | -0.08 | **0.48** | -0.06 |  | 0.23 | 0.12 | -0.02 | **0.52** | -0.12 |  | 0.25 | 0.12 | -0.05 | **0.51** | -0.13 |
| 97 | 0.18 | -0.01 | -0.02 | **0.46** | 0.11 |  | 0.14 | -0.01 | 0.06 | **0.56** | 0.05 |  | 0.14 | -0.02 | 0.07 | **0.57** | 0.06 |
| 8 | 0.21 | 0.03 | 0.06 | **0.46** | -0.07 |  | 0.20 | -0.02 | 0.12 | **0.53** | -0.11 |  | 0.22 | -0.01 | 0.10 | **0.51** | -0.11 |
| 77 | 0.13 | 0.05 | 0.12 | **0.45** | -0.07 |  | 0.10 | 0.04 | 0.17 | **0.51** | -0.15 |  | 0.10 | 0.04 | 0.18 | **0.52** | -0.14 |
| 11 | 0.25 | 0.17 | -0.06 | **0.41** | -0.01 |  | 0.22 | 0.12 | 0.00 | **0.54** | -0.02 |  | 0.23 | 0.13 | 0.00 | **0.53** | -0.01 |
| 42 | 0.00 | 0.33 | -0.16 | **0.38** | -0.08 |  | -0.04 | 0.24 | -0.12 | **0.49** | -0.11 |  | -0.05 | 0.23 | -0.12 | **0.50** | -0.12 |
| 3 | 0.04 | 0.29 | -0.19 | **0.37** | -0.26 |  | 0.04 | 0.26 | -0.15 | **0.40** | -0.23 |  | 0.06 | 0.26 | -0.17 | **0.39** | -0.24 |
| 67 | 0.14 | 0.04 | 0.19 | **0.36** | 0.15 |  | 0.11 | 0.01 | 0.24 | **0.43** | 0.14 |  | 0.10 | 0.01 | 0.25 | **0.45** | 0.15 |
| 91 | 0.28 | 0.39 | -0.27 | **0.34** | -0.08 |  | 0.26 | 0.34 | -0.22 | **0.43** | -0.04 |  | 0.26 | 0.34 | -0.25 | **0.43** | -0.04 |
| 36 | 0.29 | 0.38 | -0.23 | **0.25** | -0.22 |  | — | — | — | — | — |  | — | — | — | — | — |
| 47 | 0.08 | 0.15 | 0.07 | **0.25** | -0.03 |  | — | — | — | — | — |  | — | — | — | — | — |
| 16 | -0.09 | -0.08 | -0.36 | **0.22** | 0.09 |  | — | — | — | — | — |  | — | — | — | — | — |
| 86 | -0.18 | -0.44 | -0.15 | **0.18** | 0.09 |  | — | — | — | — | — |  | — | — | — | — | — |
| 57 | -0.10 | -0.14 | -0.31 | **0.10** | 0.05 |  | — | — | — | — | — |  | — | — | — | — | — |
| 31 | -0.23 | -0.34 | -0.04 | **0.09** | 0.19 |  | — | — | — | — | — |  | — | — | — | — | — |
| 62 | -0.22 | -0.20 | -0.17 | **-0.07** | -0.03 |  | — | — | — | — | — |  | — | — | — | — | — |
| 21 | -0.17 | -0.14 | -0.06 | **0.02** | 0.07 |  | — | — | — | — | — |  | — | — | — | — | — |
|  |  |  |  |  |  |  |  |  |  |  |  |  |  |  |  |  |  |
| 83 | 0.21 | **0.56** | 0.06 | -0.08 | -0.08 |  | 0.18 | **0.66** | 0.03 | -0.08 | -0.08 |  | 0.18 | **0.66** | 0.03 | -0.08 | -0.07 |
| 13 | 0.06 | **0.56** | -0.02 | 0.08 | -0.06 |  | 0.03 | **0.61** | 0.00 | 0.14 | -0.02 |  | 0.03 | **0.62** | -0.01 | 0.13 | -0.01 |
| 33 | 0.20 | **0.53** | 0.05 | -0.11 | -0.10 |  | 0.17 | **0.62** | 0.01 | -0.09 | -0.05 |  | 0.17 | **0.62** | 0.00 | -0.09 | -0.05 |
| 54 | 0.08 | **0.48** | 0.08 | 0.18 | -0.07 |  | 0.08 | **0.60** | 0.10 | 0.12 | -0.05 |  | 0.08 | **0.60** | 0.09 | 0.13 | -0.04 |
| 59 | -0.03 | **0.48** | 0.15 | 0.10 | 0.01 |  | -0.05 | **0.57** | 0.17 | 0.07 | 0.02 |  | -0.06 | **0.57** | 0.17 | 0.08 | 0.02 |
| 18 | 0.13 | **0.46** | 0.16 | -0.07 | -0.12 |  | 0.10 | **0.49** | 0.17 | -0.05 | -0.10 |  | 0.12 | **0.51** | 0.15 | -0.07 | -0.09 |
| 74 | -0.06 | **0.43** | 0.07 | 0.12 | 0.05 |  | -0.09 | **0.45** | 0.06 | 0.16 | 0.05 |  | -0.12 | **0.44** | 0.08 | 0.18 | 0.04 |
| 64 | 0.06 | **0.37** | 0.13 | 0.19 | -0.11 |  | 0.07 | **0.40** | 0.16 | 0.14 | -0.11 |  | 0.06 | **0.40** | 0.16 | 0.15 | -0.11 |
| 28 | -0.08 | **0.36** | 0.35 | 0.02 | -0.01 |  | -0.08 | **0.38** | 0.41 | -0.02 | -0.01 |  | -0.07 | **0.38** | 0.38 | -0.02 | -0.01 |
| 5 | -0.01 | **0.31** | -0.08 | 0.10 | -0.04 |  | -0.04 | **0.30** | -0.04 | 0.17 | -0.05 |  | -0.04 | **0.30** | -0.06 | 0.16 | -0.06 |
| 94 | 0.09 | **0.25** | 0.38 | -0.09 | -0.01 |  | — | — | — | — | — |  | — | — | — | — | — |
| 99 | 0.35 | **0.19** | 0.02 | 0.27 | 0.07 |  | — | — | — | — | — |  | — | — | — | — | — |
| 44 | 0.02 | **0.08** | -0.14 | -0.38 | 0.16 |  | — | — | — | — | — |  | — | — | — | — | — |
| 88 | -0.12 | **-0.04** | -0.39 | -0.10 | 0.13 |  | — | — | — | — | — |  | — | — | — | — | — |
| 49 | -0.05 | **-0.01** | -0.46 | -0.09 | 0.03 |  | — | — | — | — | — |  | — | — | — | — | — |
| 23 | -0.31 | **-0.01** | 0.04 | -0.27 | 0.23 |  | — | — | — | — | — |  | — | — | — | — | — |
| 38 | -0.25 | **0.00** | -0.03 | -0.22 | 0.38 |  | — | — | — | — | — |  | — | — | — | — | — |
|  |  |  |  |  |  |  |  |  |  |  |  |  |  |  |  |  |  |
| 68 | -0.20 | -0.09 | -0.08 | -0.06 | **0.63** |  | -0.20 | -0.10 | -0.02 | -0.04 | **0.69** |  | -0.20 | -0.09 | -0.03 | -0.04 | **0.69** |
| 12 | -0.07 | -0.13 | -0.04 | -0.06 | **0.61** |  | -0.06 | -0.12 | 0.01 | -0.07 | **0.66** |  | -0.07 | -0.13 | 0.02 | -0.06 | **0.66** |
| 87 | -0.10 | -0.16 | -0.07 | -0.05 | **0.55** |  | -0.09 | -0.18 | 0.00 | -0.06 | **0.62** |  | -0.09 | -0.17 | -0.02 | -0.07 | **0.63** |
| 17 | -0.03 | 0.02 | -0.17 | -0.12 | **0.49** |  | -0.04 | 0.03 | -0.14 | -0.09 | **0.56** |  | -0.03 | 0.04 | -0.16 | -0.10 | **0.57** |
| 63 | -0.29 | 0.01 | -0.34 | -0.04 | **0.35** |  | -0.30 | -0.01 | -0.31 | 0.02 | **0.43** |  | -0.30 | -0.01 | -0.33 | 0.01 | **0.43** |
| 37 | 0.00 | 0.03 | 0.01 | -0.28 | **0.34** |  | -0.01 | 0.03 | 0.00 | -0.29 | **0.31** |  | 0.03 | 0.12 | 0.36 | 0.08 | **0.32** |
| 22 | -0.01 | 0.17 | 0.36 | 0.12 | **0.31** |  | 0.01 | 0.11 | 0.40 | 0.08 | **0.32** |  | 0.00 | 0.03 | -0.01 | -0.30 | **0.32** |
| 43 | -0.44 | -0.01 | -0.03 | -0.10 | **0.31** |  | ^b^ *-0.44* | *-0.04* | *-0.01* | *-0.12* | ***0.25*** |  | — | — | — | — | — |
| 78 | -0.04 | 0.56 | 0.11 | 0.10 | **0.29** |  | — | — | — | — | — |  | — | — | — | — | — |
| 48 | -0.01 | 0.45 | 0.24 | 0.01 | **0.24** |  | — | — | — | — | — |  | — | — | — | — | — |
| 82 | -0.01 | 0.33 | 0.23 | 0.11 | **0.20** |  | — | — | — | — | — |  | — | — | — | — | — |
| 98 | -0.03 | 0.51 | 0.18 | 0.05 | **0.19** |  | — | — | — | — | — |  | — | — | — | — | — |
| 9 | -0.01 | 0.33 | -0.06 | 0.28 | **-0.19** |  | — | — | — | — | — |  | — | — | — | — | — |
| 53 | 0.13 | 0.50 | -0.04 | 0.19 | **-0.14** |  | — | — | — | — | — |  | — | — | — | — | — |
| 27 | -0.11 | 0.47 | -0.10 | 0.33 | **-0.11** |  | — | — | — | — | — |  | — | — | — | — | — |
| 92 | 0.16 | 0.21 | 0.20 | 0.24 | **0.10** |  | — | — | — | — | — |  | — | — | — | — | — |
| 58 | 0.17 | 0.29 | -0.06 | 0.28 | **0.09** |  | — | — | — | — | — |  | — | — | — | — | — |
| %^d^ | 6.77 | 6.58 | 5.22 | 5.09 | 3.60 |  | 10.41 | 6.80 | 6.07 | 5.56 | 4.91 |  | 10.23 | 7.23 | 5.86 | 5.83 | 5.07 |

F-1: N-Anx; F-2: Act; F-3: ImpSS; F-4: Agg-Host; F-5: Sy.

^a^ Largest loading for each scale is in boldface.

^b^ Item 43 was deleted because loading < 0.30 in second EFA.

^c^ Deleted items with MIs >50 after 50-item CFA.

^d^ Percentage of variance accounted for each factor. Rotation Sums of Squared Loadings.

References

Zuckerman, M., Kuhlman, D. M., Joireman, J., Teta, P., & Kraft, M. (1993). A comparison of three structural models for personality: The Big Three, the Big Five, and the Alternative Five. *Journal of Personality & Social Psychology*(65), 757-768.

Wu, Y.-X., Wang, W., Du, W.-Y., Li, J., & Wang, Y.-H. (2000). Development of a Chinese version of the Zuckerman-Kuhlman Personality Questionnaire: Reliabilities and gender/age effects. *Social Behavior and Personality: an international journal, 28*(3), 241-250.

Wang, W., Du, W., Liu, P., Liu, J., & Wang, Y. (2002). Five-factor personality measures in Chinese university students: effects of one-child policy? *Psychiatry Research, 109*(1), 37–44
